# Psychosocial functioning and determinants of the health-related quality of life in children with neurofibromatosis type 1 and cognitive impairments

**DOI:** 10.1007/s11060-025-05024-x

**Published:** 2025-06-05

**Authors:** Belinda Barton, Pamela L. Wolters, Karin S. Walsh, Nicole J. Ullrich, Tena Rosser, James Tonsgard, David Viskochil, Elizabeth Schorry, Laura J. Klesse, Michael J. Fisher, David H. Gutmann, Roger J. Packer, Bruce Korf, Maria T. Acosta, Kathryn N. North, Jonathan M. Payne, the NF Clinical Trials Consortium

**Affiliations:** 1 Faculty of Health, Southern Cross University, Coffs Harbour, NSW Australia; 2 Children’s Hospital Education Research Institute, The Children’s Hospital at Westmead, Sydney, NSW Australia; 3 Pediatric Oncology Branch, Center for Cancer Research, National Cancer Institute, National Institutes of Health, Bethesda, MD USA; 4 Center for Neuroscience and Behavioral Medicine, Division of Neuropsychology, Children’s National Hospital, Washington, DC USA; 5 The George Washington University School of Medicine, Washington, DC USA; 6 Department of Neurology, Boston Children’s Hospital, Boston, MA USA; 7 Department of Neurology, Children’s Hospital of Los Angeles, Los Angeles, CA USA; 8 Division of Neurology, University of Chicago Medicine Comer Children’s Hospital, Chicago, IL USA; 9 Department of Genetics, University of Utah, Salt Lake City, USA; 10 Human Genetics, Cincinnati Children’s Hospital Medical Center, Cincinnati, OH USA; 11 Department of Pediatrics, University of Texas Southwestern Medical Center, Dallas, USA; 12 Division of Oncology, The Children’s Hospital of Philadelphia, Philadelphia, PA USA; 13 Department of Neurology, Washington University School of Medicine, St Louis, MO USA; 14 Gilbert Family Neurofibromatosis Institute and Brain Tumor Institute, Children’s National Hospital, Washington, DC USA; 15 Department of Genetics, University of Alabama at Birmingham, Birmingham, USA; 16 Undiagnosed Disease Program, Office of the Clinical Director, National Human Genome Research Institute, National Institute of Health, Bethesda, MD USA; 17 Center for Neuroscience and Behavioral Medicine, Children’s National Health System, Washington, USA; 18 Murdoch Children’s Research Institute, The Royal Children’s Hospital, Parkville, VIC Australia; 19 Department of Paediatrics, Faculty of Medicine, Dentistry and Health Sciences, University of Melbourne, Parkville, VIC Australia

**Keywords:** Neurofibromatosis type 1, Quality of life, Psychosocial, Children, Depression, Social stress

## Abstract

**Purpose:**

Neurofibromatosis type 1 (NF1) is an autosomal dominant genetic condition associated with cutaneous and neoplastic manifestations and other physical manifestations, as well as cognitive, psychosocial, and behavioural difficulties. NF1 negatively impacts the health-related quality of life (HRQoL) of children. There is limited evidence regarding the determinants of HRQoL of children with NF1. The aim of this study was to (i) compare the HRQoL of children with NF1 and cognitive impairments to published data of healthy children and children with cancer and (ii) identify specific determinants of child and parent-proxy reports of psychosocial HRQoL.

**Methods:**

Children with NF1 and cognitive impairments (*n* = 135, 8–15 years 11 months) and their parents completed standardized measures assessing children’s HRQoL, behavioral and emotional functioning. Children completed a brief intelligence test. Correlations and multiple linear regressions were conducted to identify determinants of psychosocial HRQoL.

**Results:**

Children with NF1 had significantly poorer HRQoL for all domains than published data of healthy children and significantly poorer HRQoL for Psychosocial Health, School and Social Functioning than published data of children with cancer. For child self-report, attention problems and increased social stress predicted their psychosocial HRQoL. For parent-proxy reports, activities of daily living and depression were significant predictors of children’s psychosocial HRQoL. Social stress and depression were the strongest predictors of Psychosocial HRQoL.

**Conclusion:**

Routine screening and early identification of depressive symptoms and interventions that promote social support, coping and resiliency may improve the HRQoL of children with NF1.

**Supplementary Information:**

The online version contains supplementary material available at 10.1007/s11060-025-05024-x.

## Introduction

Neurofibromatosis type 1 (NF1) is an autosomal dominant genetic condition caused by pathogenic variants in the *NF1* tumor suppressor gene [1]. NF1 is associated with an increased risk of benign and malignant tumors [1], along with physical, cognitive, psychosocial, and behavioural difficulties [2–5].

Children with NF1, aged 8 to 16 years, have significantly poorer overall health-related quality of life (HRQoL) compared with normative data and healthy controls [6–8]. The only study comparing overall HRQoL in children with NF1 to that of other chronic conditions found that their HRQoL was similar to children with various chronic illnesses (e.g., atopic dermatosis), but significantly worse than those with asthma [6]. Children with NF1 also have poorer HRQoL in emotional, social, physical, and school domains when compared to normative data and healthy controls, although findings vary based on the generic HRQoL measure used, and between self- and parent-proxy reports [7–10].

Despite the significant impact of NF1 on HRQoL, research on its determinants in children is limited. Generally, demographic factors like age, socioeconomic status, sex, disease severity, and IQ scores do not significantly predict HRQoL [6, 9–13]. Emotional and behavioral functioning appear to be more consistent predictors. One study examined only composite scores for self-reported emotional symptoms and parent-proxy reports for behavioral symptoms, which were significant predictors of overall HRQoL [12]. Another study found that higher levels of depression and victimization reported by children predicted poorer psychosocial HRQoL [14].

Studies investigating factors contributing to the HRQoL of children with NF1 have not formally employed a conceptual HRQoL framework. One commonly used model [15] proposes that a unidirectional association exists among five health domains: biological function, symptoms, functional status, and general health perceptions, which ultimately contribute to HRQoL. In addition, individual and environmental characteristics are unidirectionally associated with each domain. Minor revisions to this model made by Ferrans and colleagues [16] were evaluated to have a better capacity to inform research and practice [17]. While both models have been widely used to examine HRQoL in chronic diseases [18], they have not been applied to NF1.

Further research is needed to identify determinants of the HRQoL in children with NF1, and the use of domain (versus composite) scores will provide additional insight into specific areas that can be targeted for interventions. We previously reported that parent-reported Attention-Deficit Hyperactivity Disorder (ADHD) symptoms were significantly associated with parent-proxy overall HRQoL of children with NF1 and cognitive impairments (NF1 + CI) [19]. The current study aimed to extend these findings by (i) comparing the HRQoL of children with NF1 + CI to published data of healthy children and children with cancer and (ii) identifying specific determinants of child and parent-proxy reports of psychosocial HRQoL.

Previous research has shown that children with cancer often experience poorer HRQoL when compared to healthy controls, particularly in physical and psychosocial domains. Factors that contribute to reduced HRQoL in pediatric oncology populations include cancer type, prognosis, treatment status (on-treatment versus off-treatment), treatment intensity, fatigue, pain, and the disruption of daily life through frequent school absences and social isolation [20, 21]. In addition, the emotional burden on families coping with a life-threatening illness and intensive medical regimens can further impact children’s psychosocial well-being [22].

We hypothesized that (i) children with NF1 + CI will exhibit significantly poorer HRQoL compared to published data for healthy children but better HRQoL compared to children with cancer, and (ii) based on the above study findings and elements of the Ferrans et al. [16] model, individual characteristics (i.e., age, sex, and intellectual functioning) will not be significant predictors but functional status (e.g., activities of daily living (ADL) and symptoms (e.g., emotions and behaviors) will be significant predictors of psychosocial HRQoL.

## Method

### Participants

Baseline data were analyzed from the STARS Phase II lovastatin multi-center randomized clinical trial (*N* = 146) [23]. For this sub-study, only children whose self-reported HRQoL was completed at baseline were included (*N* = 135). All child participants satisfied the National Institutes of Health diagnostic criteria for NF1 [24], between 8 and 15 years 11 months old at the time of screening and had impaired performance (≥ 1 SD below the population mean) on at least one of the primary cognitive outcomes measured in the STARS trial: visual-spatial learning assessed by total errors adjusted from the Paired Associate Learning (PAL) [25] or sustained attention assessed by Score [26]. The exclusion criteria for the STARS trial included Full-Scale IQ < 70, symptomatic central nervous system pathology, significantly impaired vision/hearing, insufficient comprehension of English, low baseline total cholesterol (< 90 mg/dL), and contraindications to lovastatin [23]. Disease severity and visibility, and other non-cognitive complications of NF1, were not collected.

### Measures

#### Demographic information

The race and sex of participants were collected at the time of study enrolment.

#### Health-related quality of life

The Paediatric Quality of Life Inventory (PedsQL) Version 4.0 Generic Core Scales [27] was used to assess domains of HRQoL for physical, emotional, social and school functioning. The Total Scale Score, the computed mean of all 23 items, represents the overall generic HRQoL. The Psychosocial Health Summary Score is the calculated mean of the Emotional, Social, and School Functioning Scales. Scores range from 0 to 100, with a higher score indicating better HRQoL. Self and parent proxy reports (standard version) for children (aged 8–12 years) and adolescents (13–18 years) were completed. For missing data, when 50% or more items were completed, the mean of the scale was imputed as per the scoring instructions [28]. The Peds QL is also recommended by the Response Evaluation in Neurofibromatosis and Schwannomatosis (REiNS) International Collaboration as the highest-rated generic HRQoL measure for use as an outcome measure for clinical trials in children with NF1 [29].

#### Behavioral and emotional functioning

The Behavioral Assessment System for Children– Second Edition (BASC-2) [30] the self-report (BASC-2-SRP) and parent proxy-report (BASC-2-PRS) forms assessed the emotional and behavioral functioning of children with NF1. For clinical scales (e.g., Anxiety, Hyperactivity), higher scores represent more problematic behaviors, with T-scores (M = 50, SD = 10) between 60 and 69 considered at-risk (AR) and 70 or above being clinically significant (CS). On adaptive scales (e.g., Adaptability, Social Skills), lower scores indicate deficits, with T-scores between 31 and 40 falling in the AR range and T-scores less than 31 considered CS.

The BASC-2 subscale scores rather than composite scores were used for greater specificity in predicting HRQoL. Subscales selected as potential predictors were based on elements of Ferrans’s HRQoL model [16] previous HRQoL studies of NF1 and commonly reported behavioral problems in children with NF1 (see Statistical Analysis).

#### Intellectual functioning

Four subtests (Vocabulary, Similarities, Block Design, Matrix Reasoning) from the Wechsler Abbreviated Scale of Intelligence (WASI) [31] were administered to generate an estimated Full-Scale IQ.

### Statistical analysis

Data were analyzed using the Statistical Package for the Social Sciences (SPSS) Version 28.0.1.1. Mean (M) and standard deviation (SD) or median (Mdn) and interquartile range (IQR) are reported for normal or non-normal distributed data, respectively. Cronbach’s alpha was calculated to determine the internal consistency of self- and proxy-report PedsQL scores. To assess the level of agreement between self- and parent-proxy PedsQL scores, intra-class correlation (ICC) coefficients were calculated using a 2-way mixed model, single measure with absolute agreement. ICC values and their 95% confidence intervals were classified according to guidelines [32].

PedsQL self- and proxy-report scores were compared to published data of healthy children [33] and children with cancer (50% acute lymphoblastic leukemia) who were on/off treatment [34] using independent samples t-tests. PedsQL scores that were not normally distributed were compared to published data using one-sample Wilcoxon Signed Rank tests. Effect sizes were calculated using Hedges’ *g* between NF1 + CI and healthy children, as it accounts for unequal sample sizes by incorporating pooled weighted standard deviations, providing a more accurate estimate; Cohen’s *d*, which uses the pooled standard deviation, was calculated for the NF1 + CI and cancer group comparisons, as the sample sizes were comparable; or Pearson’s *r* converted to Cohen’s *d* and classified according to Cohen’s guidelines [35].

The PedsQL Psychosocial Health summary score was used as the primary HRQoL measure. Incorporating Ferrans’s et al. [16] model, age, sex and IQ reflected individual characteristics. BASC-2 SRP and PRS subscales for Depression, Anxiety, Hyperactivity, Attentional Problems; the BASC-2 SRP subscales for Social Stress, Relations with Parents, Interpersonal Relations, Self-esteem, and Sense of Inadequacy; and the BASC-2 PRS Conduct Problems, Withdrawal, and Somatization reflected symptoms. BASC-2 PRS subscales of Adaptability, ADL, Functional Communication and Social Skills reflected functional status. Data for the Somatization scale was available only for adolescent participants (46%) and was omitted.

Pearson’s or Kendall’s tau-b correlations were conducted between age, IQ, selected BASC-2 and PedsQL Psychosocial Health scores separately for self- and parent-proxy ratings. Given the discrepancies often observed between child and parent reports of HRQoL [9, 10, 36] and to minimize potential bias arising from cross-informant differences, we conducted separate multiple linear regression analyses for each informant group. Child-reported variables were used to predict self-reported PedsQL Psychosocial Health scores, while parent-reported variables served as predictors for parent-proxy outcomes. Only variables with at least moderate strength of association with Psychosocial Health PedsQL scores (Pearson *r* ≥.4 or Kendall’s tau-b values ≥ 0.26) were included in the regression model. All tests were two-tailed, and the significance level was equal to 0.05.

## Results

### Participants

The median Full Scale IQ and age of the sample (80 males, 55 females) was 89 (IQR = 18) and 11.77 years (IQR = 3.73), respectively. The race of participants were White (81%), Black (12%), Asian (4%), other (1%) and unknown (2%), and 16% (22/135) were on a stable dose of stimulant medication.

### Health-related quality of life (HRQoL)

For one self-report, as less than 50% of the items were missing for Emotional Functioning, the mean for this scale was imputed. All PedsQL scales in the present study demonstrated acceptable internal consistency, with Cronbach’s alphas ranging from 0.631 (School Functioning) to 0.875 (Total Score) for self-report and from 0.755 (School Functioning) to 0.894 (Total Score) for parent proxy report. The level of parent-child agreement ranged from poor to moderate reliability for the Total Score (ICC: 0.36, 95% CI 0.21 to 0.50), Physical Health (ICC: 0.38, 95% CI 0.23 to 0.52), Psychosocial Health (ICC: 0.34, 95% CI 0.18 to 0.48), Emotional Functioning (ICC: 0.25, 95% CI 0.09 to 0.40), Social Functioning (ICC: 0.41, 95% CI 0.26 to 0.54), and School Functioning (ICC: 0.36, 95% CI 0.21 to 0.50). Summary scores between self- and parent-proxy reports were comparable except for Social Functioning, where children reported better HRQoL than their parents.

Children with NF1 + CI had significantly poorer self- and parent-reported HRQoL across all domains than a published sample of healthy children. Effect sizes ranged from medium to large, with the largest effect sizes for School Functioning and Psychosocial health (Table 1).

**Table 1:** Standardised PedsQL 4.0 generic scores for children with NF1 + CI compared to published children samples: healthy and cancer

PedsQL domain	NF1 + CI			Healthy sample			Cancer			NF1 + CI vs. Healthy sample		NF1 + CI vs. Cancer	
	*n*	M	SD	*n*	M	SD	*n*	M	SD	t	Hedges’ g^a^	t	Cohen’s d^a^
Child report													
Total score	135	68.56	15.60	5480	83.84	12.65	219	72.20	16.38	13.78***	1.20	2.35*	0.23
Physical health	135	78.13^b^	28.10^c^	5470	87.53	13.50	219	71.79	21.80	-6.92***^d^	0.60^e^	2.78**^d^	0.24^e^
Psychosocial health	135	65.12	16.80	5469	81.87	14.09	219	72.62	16.41	13.58***	1.18	4.14***	0.45
Emotional functioning	135	70.00^b^	35.00^c^	5468	79.33	18.15	219	71.83	21.44	-4.87***^d^	0.42^e^	-1.37^d^	0.12^e^
Social functioning	135	75.00^b^	35.00^c^	5455	85.15	16.76	219	76.84	20.31	-6.01***^d^	0.52^e^	-1.86^d^	0.16^e^
School functioning	135	55.89	18.60	5412	81.12	16.45	191	68.51	19.72	17.54***	1.53	5.83***	0.66
Parent report													
Total score	135	67.07	16.20	9430	82.70	15.40	336	69.70	19.17	11.70***	1.01	1.41	0.15
Physical health	135	78.13^b^	31.25^c^	9413	84.48	19.51	336	68.75	24.98	-4.88***^d^	0.42^e^	3.20**^d^	0.27^e^
Psychosocial health	135	63.31	16.83	9431	81.65	15.22	336	70.31	17.96	13.88***	1.20	3.89***	0.40
Emotional functioning	135	69.30	20.30	9410	81.31	16.50	336	67.53	20.32	8.37***	0.73	0.86	0.09
Social functioning	135	65.64	23.06	9406	83.70	19.43	336	75.64	20.61	10.69***	0.93	4.60***	0.46
School functioning	135	54.98	19.50	7898	78.83	19.59	250	66.40	23.19	14.03***	1.22	4.87***	0.53

Abbreviations: M = mean; SD = standard deviation; PedsQL = Pediatric Quality of Life Inventory; CI = cognitive impairment

Published data for the healthy sample from Varni et al. (2007) and for cancer from Varni et al. (2002)

Higher scores indicate better HRQoL

^a^ Effect sizes: Cohen’s *d* and Hedges’ *g*: small = 0.20, medium = 0.50, large ≥ 0.80

^b^Mdn ^c^IQR ^d^One sample Wilcoxon Signed Rank Test z statistic ^e^Effect size Pearson’s *r*: small = 0.10, medium = 0.30 and large = 0.50

*p <.05; **p <.01; *** < 0.001

When compared to a published sample of children with cancer, self-report for overall HRQoL was significantly lower for children with NF1 + CI with a small effect size, with no difference between groups for parent-proxy reports. However, there were some notable differences for specific HRQoL domains. Children with NF1 + CI experienced significantly poorer HRQoL for Psychosocial Health and School Functioning for self- and parent-proxy reports and Social Functioning for parent-proxy reports. Children with NF1 + CI had significantly better HRQoL for Physical Health self- and parent-proxy reports when compared to children with cancer. Effect sizes ranged from small to medium (Table 1).

### Behavioral and emotional functioning

Summary scores for all subscales were within normal limits except for the BASC-2-PRS Attention Problems, which was just within the AR range (Table 2). Within the BASC-2-SRP subscales, the highest percentage of children falling in the AR and CS range was for Attention Problems (43%) and Sense of Inadequacy (11%), respectively (Table 2). For BASC-2-PRS subscales, the highest percentage of children falling in the AR range was for Attention Problems (31%) and Functional Communication (31%). The highest percentage in the CS range was ADL (23%).

**Table 2:** Descriptive statistics and percentage of at-risk and clinically significant T scores for selected subscales on the behavioral assessment for children, second edition child Self-Report and Parent-Proxy report

Subscale	Self-report (*n* = 131)				Parent rating (*n* = 131)			
	Mdn	IQR	At-risk	Clinically significant	M	SD	At-risk	Clinically significant
			n (%)	n (%)			n (%)	n (%)
Depression	46.00^a^	12.00	20 (15)^a^	6 (5)^a^	52.00^b^	17.00^c^	13 (10)	16 (12)
Anxiety	50.00	16.00	23 (18)	4 (3)	54.61	12.74	21 (16)	21 (16)
Hyperactivity	51.00	16.00	26 (20)	11 (8)	57.32	13.24	22 (17)	26 (20)
Attention problems	54.00	18.00	56 (43)	13 (10)	61.00^b^	16.00^c^	41 (31)	17 (13)
Social Stress	46.00	14.00	7 (5)	8 (6)	-	-	-	-
Relations with parents^d^	52.00	14.00	16 (12)	5 (4)	-	-	-	-
Interpersonal relations^d^	55.00	14.00	10 (8)	12 (9)	-	-	-	-
Self-esteem^d^	53.00	11.00	9 (7)	5 (4)	-	-	-	-
Sense of inadequacy	53.00	17.00	8 (6)	14 (11)	-	-	-	-
Conduct problems	-	-	-	-	48.00^b^	16.00^c^	17 (13)	6 (5)
Withdrawal	-	-	-	-	54.31^a^	12.04	32 (24)	12 (9)
Adaptability^d^	-	-	-	-	49.07	10.55	22 (17)	5 (4)
Activities of daily living^d^	-	-	-	-	40.15^a^	11.51	36 (27)	30 (23)
Functional communication^d^	-	-	-	-	41.37	11.59	40 (31)	25 (19)
Social skills^d^	-	-	-	-	49.07	11.09	22 (17)	7 (5)

Note: ^a^*n* = 130 Four children and parents did not complete the BASC-2

^b^Mdn ^c^IQR

^d^Low scores on adaptive scales represent poorer functioning

T scores are reported standard mean (deviation) = 50 (10)

Abbreviations: M, mean; SD, standard deviation; Mdn, median; IQR, interquartile range

### Predictors of child-self and parent-proxy reports of HRQoL psychosocial health

PedsQL Psychosocial Health child self-report scores correlation values ranged from negligible (Full-Scale IQ) to strong (BASC-2-SRP Social Stress) (Table S1). Psychosocial Health self-report scores correlated moderately or higher with BASC-2-SRP subscales for Depression, Anxiety, Attention Problems, Social Stress, Interpersonal Relations, and Sense of Inadequacy. BASC-2-SRP Attention Problems and Social Stress subscales were significant predictors of PedsQL Psychosocial Health self-report, accounting for 59% of the variance in scores (Table 3), with Social Stress being the strongest predictor.

**Table 3:** Regression coefficients for predicting children’s PedsQL psychosocial health (Self-Report)

Variable	B	95% CI	β	t	*p*
Intercept	127.57	[98.71, 156.24]	-	8.81	< 0.001***
Sex	1.94	[-2.08, 5.95]	0.06	0.96	0.34
BASC-2-SRP Depression	-0.04	[-0.33, 0.26]	-0.02	-0.24	0.81
BASC-2-SRP Anxiety	-0.12	[-0.33, 0.15]	-0.07	-0.89	0.37
BASC-2-SRP Hyperactivity	0.06	[-0.17, 0.28]	0.04	0.50	0.62
BASC-2-SRP Attention Problems	-0.29	[-0.54, -0.05]	-0.18	-2.41	0.02*
BASC-2-SRP Social Stress	-0.97	[-1.30, -0.64]	-0.59	-5.78	< 0.001***
BASC-2-SRP Interpersonal Relations	0.08	[0.19, 0.36]	0.05	0.60	0.55
BASC-2-SRP Sense of Inadequacy	-0.01	[-0.24, 0.23]	-0.01	-0.06	0.96

Note. R^2^_adj_ = 0.59 (*N* = 130, *p* <.001). CI = confidence interval for B

*p <.05; **p <.01; ***p <.001

PedsQL Psychosocial Health parent-proxy scores correlation values ranged from negligible (Full-Scale IQ) to strong (BASC-2-PRS Depression) (Table S2). Psychosocial Health parent-proxy scores correlated moderately or higher with the BASC-2-PRS subscales for Depression, Hyperactivity, Attention Problems, Withdrawal, Adaptability, ADL, and Functional Communication. BASC-2-PRS Depression and ADL were significant predictors of PedsQL Psychosocial Health parent-proxy report scores, accounting for 55% of the variance in scores (Table 4), with Depression being the strongest predictor.

**Table 4:** Regression coefficients for predicting children’s PedsQL psychosocial health (Parent-Proxy)

Variable	B	95%CI	β	t	*p*
Intercept	91.12	[65.45, 116.78]	-	7.03	< 0.001***
Sex	2.97	[-1.24, 7.18]	0.09	1.40	0.17
BASC-2-PRS Depression	-0.63	[-0.87, -0.39]	-0.45	-5.25	< 0.001***
BASC-2-PRS Hyperactivity	-0.14	[-0.35, 0.07]	-0.11	-1.35	0.18
BASC-2-PRS Withdrawal	-0.10	[-0.31, 0.11]	-0.07	-0.99	0.33
BASC-2-PRS Adaptability	0.02	[-0.23, 0.27]	0.02	0.18	0.86
BASC-2-PRS Activities of daily living	0.51	[0.23, 0.79]	0.35	3.59	< 0.001***
BASC-2-PRS Functional communication	-0.08	[0.56, -0.36]	-0.05	-0.59	0.56

Note. R^2^_adj_ = 0.55 (*N* = 130, *p* <.001). CI = confidence interval for B

*p <.05; **p <.01; ***p <.001

## Discussion

This study examined the child self- and parent-proxy reports of HRQoL of children with NF1 with documented cognitive impairments compared to published data for healthy children and children with cancer, as well as identified predictors of psychosocial HRQoL. Consistent with previous studies [7, 9], both self- and parent-proxy reports indicated significantly poorer HRQoL overall and for all domains than healthy children. The most significant disparity for HRQoL between children with NF1 + CI and published data was for self and parent-proxy reports of school functioning. However, the disparity was greater than in previous studies [7, 8, 11, 13]. The School Functioning Scale measures difficulties in paying attention, forgetting things, and keeping up with school. Participants likely experienced greater impairment in school functioning due to the study inclusion criterion of impaired performance on attention [23].

Self- and parent-proxy reports of HRQoL of children with NF1 + CI were significantly poorer than children with cancer for psychosocial health and school functioning and also for parent-proxy reports of social functioning. Conversely, the HRQoL for physical health of children with NF1 + CI was significantly better than children with cancer, half of whom were receiving medical treatment for their cancer, which may account for this difference. These findings suggest that children with NF1 + CI face unique challenges in their psychosocial functioning compared to their peers with cancer, despite having better HRQoL for physical health. Unlike cancer, which often follows a more time-limited illness trajectory, children with NF1 + CI have a lifelong, progressive condition. The persistence of cognitive and attentional impairments, social difficulties, and the absence of a clear recovery period may contribute to a cumulative psychosocial burden. These ongoing challenges may not only affect children’s emotional and social well-being but also place a strain on family functioning and children’s ability to engage in everyday activities like school and socialization.

Consistent with our hypotheses, age, sex, and IQ were not significantly associated, while functional status and symptoms were significantly associated with self and parent-proxy reports for Psychosocial HRQoL. For self-report, attention problems and social stress predicted their psychosocial HRQoL. This finding is somewhat consistent with a previous study of adolescents with NF1 and plexiform neurofibromas with daily pain, where social stress and pain interference were significant predictors of overall poorer HRQoL [37]. In the current study, social stress was the strongest predictor. The Social Stress scale reflects the pervasive stressful feelings children experience in social situations, including feeling lonely, out of place and lacking supportive friendships [38]. In addition to social challenges, attentional problems were prevalent, with around half of the children and parents reporting significant difficulties with tasks involving learning, concentration and attention. Difficulties that often co-occur with attentional problems may substantially contribute to poorer HRQoL. Children with NF1 and ADHD symptomology have poorer social skills [2, 39], which may contribute to difficulties in forming and maintaining friendships. In the general population, ADHD has a substantial negative impact on children’s overall HRQoL, which is greater for psychosocial than physical domains [40]. This is likely due to the symptoms of ADHD, associated emotional and behavioral regulation difficulties, and comorbid conditions affecting school and social functioning [41].

For parent-proxy reports, depression and ADL were significant predictors of children’s psychosocial HRQoL, with depression being stronger. Depression has been found to be a significant predictor in children [14] and adults with NF1 [42]. Around 20% of children had elevated symptoms of depression, which may be related to social withdrawal, hyperactivity, and social stress. The other predictor, ADL, reflects the child’s ability to independently perform daily living tasks, organize tasks, and act safely. Parent responses indicated that around half of the children experienced deficits in ADL. While adaptive behavior impairments are often associated with intellectual disabilities [38], all children had an IQ above 70, with no significant association between ADL and IQ scores. However, ADL was significantly associated with BASC-2-PRS Hyperactivity, Attention Problems and Functional Communication. Previous studies suggest that adaptive functioning impairments in children with NF1 are associated with attention [43, 44], suggesting that the ADL impairments are likely related to underlying attentional difficulties in this cohort [23].

## Clinical implications

Based on the strongest predictors identified, perceived social support could be critical in improving the HRQoL of children with NF1 + CI. Perceived social support is a protective factor for children’s physical and mental health outcomes, including depression and stress [45, 46]. During childhood and adolescence, support from parents and teachers are stronger and more consistent sources of social support than support from friends and others [45, 47]. Social support can directly benefit outcomes independent of stress levels and buffer the effects of high stress levels [48]. High levels of social support also promote resiliency [49]. Resilient individuals tend to utilise adaptive coping strategies, such as seeking support and problem-solving, which can help manage and minimise the negative effects of future stressors [50]. Interventions that promote social support [51, 52], adaptive coping strategies and resiliency may effectively improve the HRQoL of children with NF1. The Resilient Youth with NF, an effective intervention in improving the psychological HRQoL, coping, resiliency and social support of adolescents with neurofibromatoses [53, 54], could be modified to be age-appropriate for children with NF1. Routine screening, early identification and treatment of depressive symptoms also may lead to better HRQoL of children with NF1.

## Study limitations

A relatively large cohort of children with NF1 + CI was recruited from NF academic clinics across the US and Australia [23], however, not all components of the Ferrans et al. model [16], which may be relevant to NF1, was collected. For instance, physical symptoms such as pain, skin sensations, bothersome itching and other disease-specific symptoms such as speech difficulties and perceived cognitive functioning may have an adverse effect on the generic HRQoL of children with NF1 [55–57]. Also, the inclusion criteria of impaired attention and/or visual-spatial learning may limit generalizability, despite these difficulties being common in NF1 [3, 25]. The study’s inclusion criterion could influence the high incidence of attentional problems [38], however, it aligns with other published data and likely reflects the ADHD symptomology often observed in NF1 [3, 4, 58]. Another limitation is the requirement for participants to be English-speaking, due to the availability of some measures only in English. This may have excluded individuals from diverse linguistic backgrounds and limited the generalizability of the findings.

In this study, we analyzed child-reported and parent-reported data separately to maintain consistency between predictors and HRQoL within informant groups. This approach was chosen considering the well-documented informant discrepancies between child and parent reports, which may reflect differences in perspectives, the child’s age and the specific HRQoL domain assessed [36]. Analyzing data within informants minimizes potential confounding introduced by cross-informant variation and allows for a clearer interpretation of the predictors of HRQoL from each informant’s perspective. However, this approach presents a limitation, as it increases the potential for common method variance (CMV) whereby associations between predictors and outcomes may be inflated due to shared method effects, such as response biases.

One potential strategy to address CMV is the use of cross-informant regression models, in which predictors reported by one informant (e.g., parents) are used to predict outcomes reported by another (e.g., children), and vice versa. While such models may mitigate concerns about CMV, they introduce other complexities, given the typically low to moderate agreement between parent and child reports [59]. Cross-informant analyses may attenuate associations and obscure meaningful informant-specific relationships. Nevertheless, this is an important avenue for future research to better understand informant discrepancies and the interrelationships between parent- and child-reported outcomes.

Moreover, the predictor variables included in the self-report and parent-proxy regression models were largely distinct, with only limited overlap in the selected BASC-2 subscales (i.e., Depression, Anxiety, Hyperactivity, and Attention Problems). This distinction limits direct comparisons of the predictors of HRQoL across informants. However, previous research has consistently shown low to moderate agreement between child and parent ratings of social, emotional, and behavioral difficulties [59]. In particular, parents tend to report more severe symptomatology than children, especially regarding depression and hyperactivity [60]. These informant discrepancies underscore the rationale for analyzing parent and child reports separately in the current study and highlight the complexity of interpreting cross-informant relationships.

Finally, the cross-sectional design of this study limits the ability to determine the directionality of associations between HRQoL and behavioral/emotional functioning. It is possible that poorer HRQoL may contribute to increased behavioral and emotional difficulties, rather than these difficulties solely predicting lower HRQoL. Longitudinal research is needed to clarify the temporal relationships and potential bidirectional influences between these variables.

Additionally, future research should focus on determinants of HRQoL that are modifiable to treatment, such as the perceived social support and coping styles of children with NF1. Considering Ferrans’s model [16] and existing literature on factors that are associated with poor HRQoL in other populations, the general health perceptions of children with NF1, characteristics of their environment (e.g., family functioning, socioeconomic status, parental psychological well-being), other symptoms that occur in children with NF1 (e.g., ADHD, autism and sleep problems) could offer valuable insights. Incorporating NF1-specific disease symptoms and utilizing disease-specific HRQoL measures [61] would further enhance our understanding of the determinants of poor generic HRQoL in children with NF1. Future studies should also consider integrated analytic approaches, such as cross-informant regression models, to examine these relationships and provide a more comprehensive understanding of the factors contributing to discrepancies between parent and child reports.

## Conclusion

This study presents unique insights into the HRQoL of children with NF1 + CI. Both self- and parent-proxy reports reveal significantly poorer psychosocial HRQoL in this population compared to healthy children and those with cancer. Our results enhance the understanding of specific determinants of HRQoL, including depression, social stress, daily living activities, and attention problems. The diversity of these predictors highlights the complex interplay between psychological, social, and functional aspects of life for children with NF1. These findings underscore the urgent need for targeted interventions that address these multifaceted challenges, ultimately promoting better HRQoL of children with NF1 + CI.

## Electronic supplementary material

Below is the link to the electronic supplementary material.

Supplementary Material 1

## Data Availability

The dataset analyzed during the current study is available from the corresponding author upon reasonable request.
