# Supplementary material for: Psychosocial functioning and determinants of the health-related quality of life in children with neurofibromatosis type 1 and cognitive impairments
Source: J Neurooncol. 2025 Jun 5;174(1):65–76. doi: 10.1007/s11060-025-05024-x (PMC12198070; doi:10.1007/s11060-025-05024-x)
Supplement: Supplementary file 1 — Supplementary Material 1 [file 11060_2025_5024_MOESM1_ESM.docx]

**Supplementary Table 1**. Kendall’s tau-b Correlation Matrix between PedsQL Psychosocial (child self-report) BASC-2-SRP, age, and Full Scale IQ (n = 131).

| Variable | 1 | 2 | 3 | 4 | 5 | 6 | 7 | 8 | 9 | 10 | 11 | 12 |
| --- | --- | --- | --- | --- | --- | --- | --- | --- | --- | --- | --- | --- |
| 1. Depression | - |  |  |  |  |  |  |  |  |  |  |  |
| 2. Anxiety | .39**^a^ | - |  |  |  |  |  |  |  |  |  |  |
| 3. Hyperactivity | .18**^a^ | .37** | - |  |  |  |  |  |  |  |  |  |
| 4. Attention problems | .22**^a^ | .29** | .36** | - |  |  |  |  |  |  |  |  |
| 5. Social stress | .43**^a^ | .43** | .32** | .34** | - |  |  |  |  |  |  |  |
| 6. Relations with parents | -.24**^a^ | -.11 | -.03 | -.09 | -.15* | - |  |  |  |  |  |  |
| 7. Interpersonal relations | -.30**^a^ | -.25** | -.23** | -.33** | -.55** | .17** | - |  |  |  |  |  |
| 8. Self-esteem | -.41**^a^ | -.32** | -.11 | -.22** | -.28** | .27** | -.22** | - |  |  |  |  |
| 9. Sense of inadequacy | .42**^a^ | .37** | .25** | .41** | .37** | -.16* | .30** | -.30** | - |  |  |  |
| 10. Age | -.11^a^ | -.04 | -.10 | .11 | -.02 | .02 | -.13* | .02 | .10 | - |  |  |
| 11. Full-Scale IQ | -.13*^a^ | -.01 | .08 | .02 | .04 | -.04 | .06 | .08 | -.09 | -.09 | - |  |
| 12. PedsQL Psychosocial child self-report | -.37**^a^ | .37** | -.24** | -.34** | -.56** | .15* | .39** | .23** | -.32** | .08 | -.01 | - |

^a^n = 130

**p* < .05. ***p* <.01.

**Supplementary Table 2.** Correlation Matrix between Psychosocial HRQoL (parent proxy), BASC-2-PRS subscales, age, and Full Scale IQ (n = 131).

| Variable | 1 | 2 | 3 | 4 | 5 | 6 | 7 | 8 | 9 | 10 | 11 | 12 | 13 |
| --- | --- | --- | --- | --- | --- | --- | --- | --- | --- | --- | --- | --- | --- |
| 1. Depression | - |  |  |  |  |  |  |  |  |  |  |  |  |
| 2. Anxiety | .34**^b^ | - |  |  |  |  |  |  |  |  |  |  |  |
| 3. Hyperactivity | .40**^b^ | .19* | - |  |  |  |  |  |  |  |  |  |  |
| 4. Attention problems | .22**^b^ | .02^b^ | .49**^b^ | - |  |  |  |  |  |  |  |  |  |
| 5. Conduct problems | .29**^b^ | -.09^b^ | .40**^b^ | .25**^b^ | - |  |  |  |  |  |  |  |  |
| 6. Withdrawal | .40**^ab^ | .21*^a^ | .27**^a^ | .22**^ab^ | .07^ab^ | - |  |  |  |  |  |  |  |
| 7. Adaptability | -.37**^b^ | -.17* | -.48** | -.34**^b^ | -.26**^b^ | -.38**^a^ | - |  |  |  |  |  |  |
| 8. Activities of daily living | -.26**^ab^ | -.10^a^ | -.54**^a^ | -.55**^ab^ | -.21**^ab^ | -.38**^a^ | .57**^a^ | - |  |  |  |  |  |
| 9. Functional communication | -.23**^b^ | -.08 | -.08 | -.49**^b^ | -.21**^b^ | -.41**^a^ | .49** | .73**^a^ | - |  |  |  |  |
| 10. Social skills | -.15*^b^ | .18* | .18* | -.29** | -.19**^b^ | -.41**^a^ | .49** | .55**^a^ | .58** | - |  |  |  |
| 11. Age | .03^b^ | .05 | .05 | -.11^b^ | .00^b^ | -.02^a^ | -.06 | .00^a^ | -.07 | .02 | - |  |  |
| 12. Full-Scale IQ | -.02^b^ | -.09 | -.09 | -.03^b^ | -.10^b^ | -.02^a^ | .09 | .08^a^ | .26** | .22 | -.13 | - |  |
| 13. Peds QoL Psychosocial parent proxy | -.52**^b^ | -.37** | -.55** | -.37**^b^ | -.19**^b^ | -.45**^a^ | .49** | .55**^a^ | .42** | .22* | -.04 | .01 | - |

^a^n = 130 ^b^Kendall’s tau-b correlation coefficient

**p* < .05. ***p* <.01.
